# Cancer immunotherapy: Moving beyond checkpoint inhibition

**DOI:** 10.18632/oncotarget.26384

**Published:** 2018-11-27

**Authors:** Marianne Boes

**Affiliations:** Marianne Boes: Department of Pediatrics and Laboratory of Translational Immunology, University Medical Center Utrecht, Utrecht University, Utrecht, The Netherlands

**Keywords:** immunotherapy, MHC, cancer, immunogenicity, cytotoxic

Immunotherapy based on checkpoint blocking antibodies (anti CTLA4, anti PD-1, anti PD-L1) has improved the treatment outcome for patients suffering from cancer. Well responding tumors include melanoma and non-small cell lung cancer, which are tumors characterized by having acquired mutational loads and a relatively high expression of neoantigens. While checkpoint-based immunotherapy has revolutionized the treatment of cancer, only a fraction of patients responds and many types of cancer appear refractory altogether [1, 2]. Neuroblastoma is an example of an embryonic tumor that has low mutational load and few neoantigens, for which susceptibility to checkpoint blockade-based immunotherapy is not evident. It is derived from incompletely committed peripheral nerve precursor cells of the neural crest and accounts for approximately 8% of all childhood cancers and 15% of childhood cancer mortality. Neuroblastoma is a notoriously non-immunogenic tumor, contributed by a low level of expression of products of the major histocompatibility (MHC) Class-I locus. As a consequence, neuroblastoma tumors evade a major immune defense strategy based on MHC-restricted cytotoxic T cells [3, 4]. Since cancer immunotherapy is not restricted to checkpoint inhibition, other immune-based therapeutic avenues are under investigation for a variety of cancers, including dendritic cell vaccination [5] and adoptive T cell therapy using tumor-infiltrating lymphocytes or using gene transfer of a chimeric antigen receptor (CAR) into T cells [6]. Improving the immunogenicity of tumors usually benefits cancer recovery.

Many tumors show decreased levels of peptide/MHC-I complexes at their cell surface, either through alteration of the levels of MHC subunits themselves or through manipulation of accessory proteins in the antigen processing/presentation pathway. Considering neuroblastoma, the tumors develop from early progenitor cells that usually do not express immunological features such as MHC-I, which is conceptionally different from adult tumors that downregulate MHC-I as immune evasion strategy. Embryonic stem cells also display low levels of MHC-I molecules at their cell surface [7]. MHC-I surface display in neuroblastoma therefore should involve different mechanistic trajectories than tumors developed from healthy cells that do express MHC-I.

To improve the immunogenicity of neuroblastoma, one approach is to upregulate MHC-I display at the tumor cell surface, which requires definition of key regulatory protein targets. In neuroblastoma and other tumor cells, lowered MHC-1 expression levels correlate with a reduction in NFκB signaling [8, 9]. In healthy cells, NFκB directly transactivates gene transcription for MHC-I heavy chains as well as the β2-microglobulin (β2m) light chain. NFκB also regulates the expression of other MHC-I antigen presentation machinery gene products, including TAP1 and TAP2, ERAP1 and ERAP2 and tapasin [8]. With the laboratory of Thijn Brummelkamp, we executed a genome-wide screen using mutagenesis of a clonal neuroblastoma cell line with a CRISPR knock-out library. This approach yielded us two gene products, TNIP1 and N4BP1, NEDD4-binding protein 1 (inhibitor of the E3 ubiquitin-protein ligase ITCH) and TNIP1 (TNFAIP3-interacting-protein-1, inhibitor of NFkB activation by regulating IκB-kinase γ-subunit deubiquitination), that control peptide/MHC-I display at the neuroblastoma cell surface. Deletion of these gene products in neuroblastoma cell lines revealed increased NFκB activity with corresponding increase in MHC-I antigen presentation capacity and induced recognition by tumor-antigen specific T cells. In support, we showed that patients expressing high levels of TNIP1 and N4BP1 in neuroblastoma have lowered MHC-I tumor surface display, and have worse survival probability [10].

Our mutagenesis and screen-based approach proved useful in defining key proteins that regulate peptide/MHC-I presentation in neuroblastoma. Reduced MHC-I cell surface display of adult tumors however should involve regulatory trajectories that are distinct from those in embryonic tumors. In adult tumors, reduced MHC-I presentation is more likely the consequence of immune pressure selecting for outgrowth of transformed tumor cells with lowered immunogenicity. Embryonic tumors instead are more likely derived from precursor cells that were yet to express the MHC-I levels of fully differentiated cells [10]. Lessons were learned from research in the virus field revealing a variety of cellular target proteins that viruses use, to downregulate MHC-I and avoid immune recognition. For tumors, a broad array of MHC-I immune evasion mechanisms is also to be expected, for adult and embryonic tumors. Mutagenesis and screening-based approaches geared to discovery of regulatory proteins for peptide/MHC-I presentation should reveal new therapeutic targets, towards improving tumor immunogenicity and ultimately to the benefit of cancer immunotherapy.
